# Research on the influencing factors and improvement paths of digital trade development in the Yangtze River Economic Belt——Based on entropy-weighting TOPSIS and fsQCA method

**DOI:** 10.1371/journal.pone.0284519

**Published:** 2023-07-17

**Authors:** Xiaomei Hu, Fan Liu

**Affiliations:** School of Management Science and Engineering, Anhui University of Finance and Economics, Bengbu, Anhui Province, China; University of Belgrade Faculty of Organisational Sciences: Univerzitet u Beogradu Fakultet organizacionih nauka, SERBIA

## Abstract

While China’s digital trade is developing rapidly, it is facing the problem of uncoordinated and uneven regional development. In order to explore countermeasures to bridge the differences in regional digital trade, this paper first measures the development level of digital trade in 11 provinces of the Yangtze River Economic Belt through entropy-weighting TOPSIS. Secondly, the fuzzy set Qualitative Comparative Analysis method was used to analyze the influencing factors and improvement paths of digital trade development in various provinces of the Yangtze River Economic Belt. The research results show that: first, the current development level of digital trade is gradually weakening from east to west, especially the development of digital trade in the Yangtze River Delta region is the best, showing a large regional imbalance and uncoordinated development. What’s more, the market size and urbanization have the most significant impact on the development of digital trade. Finally, we examine the reliability of the results by sensitivity analysis, and find that our ranking results are robust. In the future, China should continue to actively promote the coordinated development strategy of regional economy and participate in international digital trade exchanges and cooperation, so as to promote the coordinated and high-quality development of digital trade.

## Introduction

With China’s application to join the Digital Economy Partnership Agreement, digital trade, as an important component of the digital economy, has attracted particular attention. The Ministry of Commerce predicts that by 2025, China’s total digital service trade imports and exports will exceed $400 billion, accounting for about 50% of total service trade. However, while China’s digital trade has made obvious progress, it is also facing the problem of unbalanced development between urban and rural areas and regions. In recent years, China’s regional development differentiation has become increasingly obvious, and the development of northwest China and other regions is facing many difficulties: the development momentum is obviously insufficient, and the development vitality has not been fully released.

The United States International Trade Commission noted that digital trade is a domestic business and international trade activity in which the Internet and Internet technologies play an important role in the delivery, production and ordering of products or services. Accordingly, China’s Ministry of Commerce believes that digital trade is the sum of services, goods and cross-border data flow trade relying on networks and digital technologies. The definition of it can be discussed from two perspectives in: broad and narrow. Tan [1] believes that digital products and digital services provided through the Internet are classified as digital trade. Xiao et al. [2] believe that digital trade refers to the digitalization of cross-border trade objects and trade methods based on information network technology. Different scholars have different views on the development model of digital trade. Cao et al. [3] proposed that it is necessary to establish and improve the data security protection system. It can be easily found that other scholars have mainly discussed the evolution path of digital trade from international perspectives. Kwak [4] provided an overview of the integration of the principle of technological neutrality in the world digital trading system. Collins et al. [5] concluded that every country must achieve the goal of equality and fairness in the transaction process through a “soft landing”. As mentioned above, most current studies analyze digital trade theoretically. Few scholars measure the development level of digital trade and analyze its influencing factors empirically.

In this study, we mainly employ Entropy-TOPSIS (approximate ideal solution ranking) model and QCA (Qualitative Comparative Analysis) to analyze the development status of digital trade in various regions. Entropy method is a relatively objective weighting method, and it is not easily affected by subjective factors [6]. However, due to various factors, this method is not completely objective [7]. So it is necessary to analyze the sensitivity of obtained result [8]. The existing treatment method is mainly to test the robustness of conclusion by changing weight coefficient [9]. Different from traditional significance tests, QCA is based on sets and can search for combinations of conditions that lead to specific results [10]. QCA can reveal a complex causal relationship, showing that a condition only works when other conditions occur [11]. In it, both conditions and outcomes are presented as sets, and their values are based on specific membership scores [12]. The Yangtze River Economic Belt is an economic belt that runs through the three major regions of China, one of the “three major strategies” implemented by the central government, and a coordinated belt with global influence for inland river economic belt and strengthening interaction and cooperation between the east, central and western regions. Therefore, the objective of this study is to evaluate digital trade in various regions of the Yangtze River Economic Belt, analyze the factors affecting the development of digital trade, and provide effective proposals to promote the development of digital trade.

There are few researches on the development level of digital trade and the main factors affecting it. So the possible contributions or innovation points of this article are as follows. First of all, entropy-weighting TOPSIS is used to measure digital trade in different regions, which is conducive to clarifying the incongruity phenomenon between regions. Secondly, through qualitative comparative analysis, the specific combination conditions leading to the unbalanced development of regional digital trade are discussed, which is conducive to further propose effective solutions to the existing problems. The study in this paper principally solves two problems, one is to measure the development level of digital trade, the other is to identify the pivotal factors affecting the development of digital trade.

The rest of the article is arranged as follows: Section 2 reviews the literature and points out the purpose and contribution of this study. Section 3 describes the research methods used in the article. Section 4 presents the research results. Section 5 is sensitivity analysis. The last section is the discussion of the research results, the shortcomings of this research as well as the direction of future improvement.

## Research methodology

### Research framework

**Fig 1:**
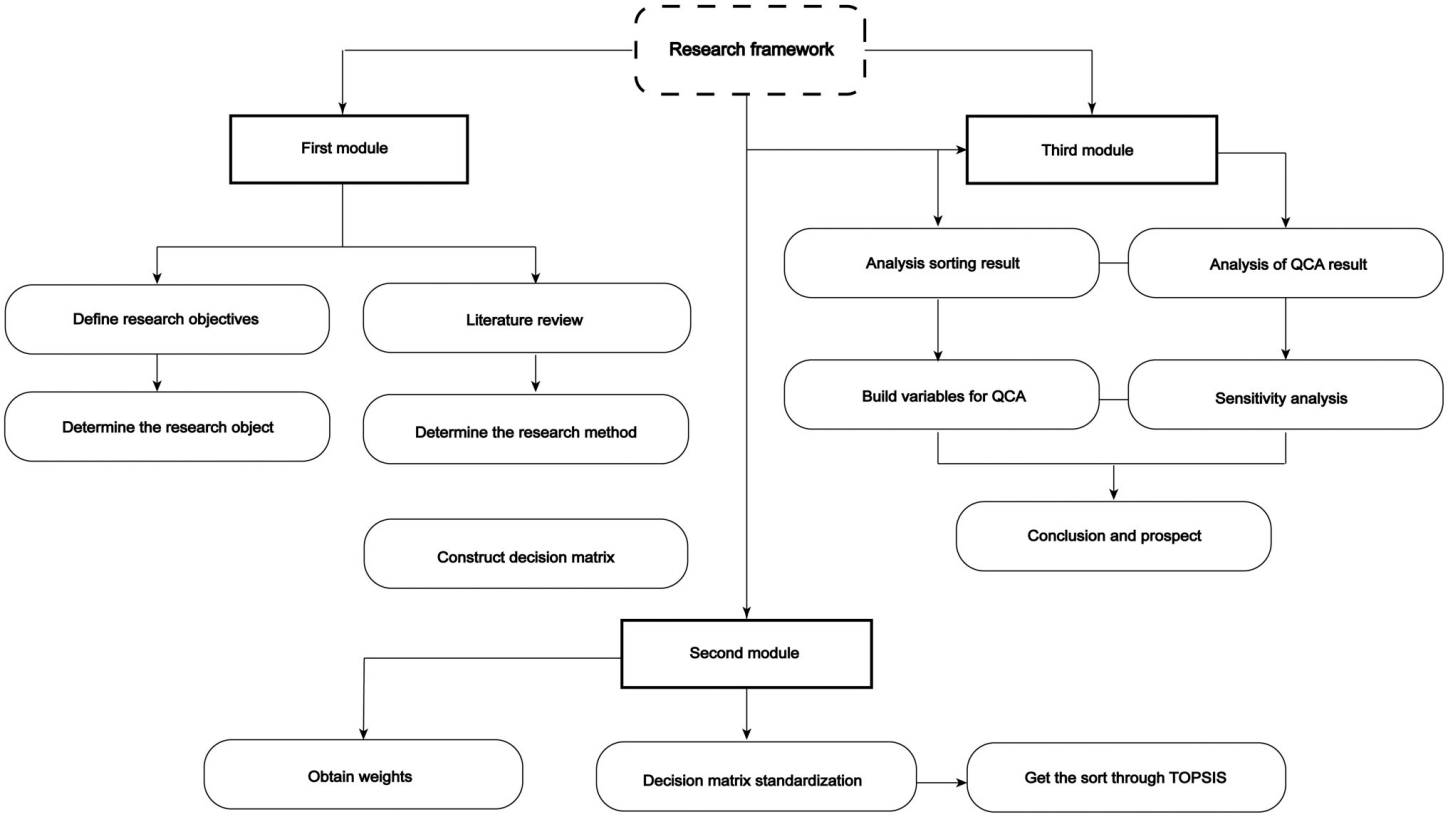
Flowchart of research methods.

The research methods in this paper are entropy-weighting TOPSIS (approximate ideal solution ranking) and QCA (Qualitative Comparative Analysis). Multi Criteria Decision Making (MCDM) methods are used to determine the weight and rank of alternatives [13]. MCDM methods are able to identify the best solution from a variety of alternatives [14]. Firstly, by constructing a digital trade evaluation system, the development level of digital trade in 11 provinces and cities in the Yangtze River Economic Belt was indirectly measured by using TOPSIS as the result variable. Secondly, in order to explore the combination of conditions that contribute to the outcome, QCA was used for further analysis. By using qualitative comparative analysis methods, the influencing factors and improvement paths of digital trade development in various provinces of the Yangtze River Economic Belt are discussed through selecting conditional variables. QCA is divided into clear set Qualitative Comparative Analysis (csQCA), multi-value set Qualitative Comparative Analysis (mvQCA) and fuzzy set Qualitative Comparative Analysis (fsQCA). Different from classical econometric statistics methods, QCA conducts conditional configuration analysis on problems with the theory of sets, relaxing many assumptions in classical quantitative analysis. Classical quantitative analysis takes ideal, direct causality as the starting point to avoid the problem of limited diversity, but QCA believes that the causal relationship between things may be complex through the idea of configuration analysis based on case studies. The framework structure of this study is shown in Fig 1.

### Entropy method and TOPSIS

TOPSIS constructs positive ideal solutions and negative ideal solutions of multi-attribute problems, and uses two benchmarks close to positive ideal solutions and far away from negative ideal solutions as evaluation criteria. When evaluating in this way, the differences in the dimensions of the indicator attributes must be taken into account, so the indicators must be reduced in dimension first. According to existing studies, it is found that the evaluation results after the standardization of each index by vector normalization method are closest to the true level when TOPSIS is used [15]. Therefore, the vector normalization method is used to standardize the evaluation indexes.

Firstly, the entropy method is used to calculate the weightings. The indicator matrix is *X* = (*x*_*ij*_)_*m×n*_ (*i* = 1, 2, 3, …,*n*), the weight vector of the indicator is *W* = (*w*_1_, *w*_2_, …, *w*_*n*_)^*T*^, where *m* indicates the number of evaluation objects, *n* indicates the number of evaluation indicators, *w*_*n*_ is the weight of each evaluation index, *X* is the decision matrix, and *x*_*ij*_ are its elements.

In the first step, the vector normalization method is used to standardize the index matrix to obtain the standardized matrix *Y*_*ij*_ = (*y*_*ij*_)_*m×n*_, where *Y* is the initial decision-making matrix, and *y*_*ij*_ are its elements. For the efficiency metric and the cost-oriented metric, the conversion formulas are shown below:

$$y_{ij}=\frac{x_{ij}}{\sqrt{\sum_{i=1}^{m}x^{2}{}_{ij}}}$$ (1)

$$y_{ij}=\frac{1/x_{ij}}{\sqrt{\sum_{i=1}^{m}{\left(1/x_{ij}\right)}^{2}}}$$ (2)

The *p*_*ij*_ of the *j* − *th* evaluation indicator of the *i* − *th* evaluation object is calculated:

$$p_{ij}=\frac{y_{ij}}{\sum_{i=1}^{m}y_{ij}}$$ (3)

Calculate the entropy *e*_*j*_ and *q*_*j*_ of every evaluation indicator:

$$e_{j}=-k\sum_{i=1}^{m}p_{ij}\times \text{ln}\;p_{ij},k=\frac{1}{\text{ln}\;m}$$ (4)

$$q_{j}=1-e_{j}$$ (5)

Calculate the weight of each evaluation indicator:

$$w_{j}=q_{j}/\sum_{j=1}^{n}q_{j}$$ (6)

Calculate the weighted decision matrix *V*, and *v*_*ij*_ are the elements of it:

$$V={\left(v_{i}{}_{j}\right)}_{m\times n}={\left(w_{j}y_{ij}\right)}_{m\times n}$$ (7)

Determine the ideal and negative ideal solutions. V* and *V*^−^ represent ideal value and non-ideal value respectively.$v_{n}{}^{*}$ represents benefit criteria and $v_{n}{}^{-}$ represents cost criteria:

$${\text{V}}^{*}=\{\underset{1\leq i\leq m}{\text{max}}v_{ij}\}=\{v_{1}{}^{*},v_{2}{}^{*},\ldots ,v_{n}{}^{*}\}$$ (8)

$${\text{V}}^{-}=\{\underset{1\leq i\leq m}{\text{min}}v_{ij}\}=\{v_{1}{}^{-},v_{2}{}^{-},\ldots ,v_{n}{}^{-}\}$$ (9)

The Euclidean distance to the positive solutions $S_{i}{}^{*}$ and negative ideal solutions $S_{i}{}^{-}$ is calculated for each object:

$$S_{i}{}^{*}=\sqrt{\sum_{j=1}^{n}{\left(v_{ij}-v_{j}{}^{*}\right)}^{2}}(i=1,2,3,\ldots ,m)$$ (10)

$$S_{i}{}^{-}=\sqrt{\sum_{j=1}^{n}{\left(v_{ij}-v_{j}{}^{-}\right)}^{2}}(i=1,2,3,\ldots ,m)$$ (11)

Calculate the relative proximity of each object, the higher the value, the better the development of digital trade in the region.


$$C_{i}{}^{*}=\frac{S_{i}{}^{-}}{S_{i}{}^{*}+S_{i}{}^{-}}$$ (12)

### Measurement metrics

This paper relies on diamond theory to construct measurement indexes of the digital trade. The diamond theory was formally proposed by the famous American scholar Michael Porter in the book “National Competitive Advantage”, which reveals the source of a country’s wealth. The diamond theory reveals various factors affecting economic growth in a specific area of a region. Diamond theory has been applied to various fields, as far as the research literature published by domestic scholars in recent years. Although the core of “National Competitive Advantage” lies in the theory of competitive strategy, it involves many fields, such as development economics, economic geography and international trade. So the study of economic growth can also be discussed in the framework of diamond theory.

Diamond theory consists mainly of four basic elements. Production factors are divided into primary and advanced production factors, and primary production factors include tangible and intangible resources such as natural climate, location conditions, and mineral resources. Advanced production factors are reflected in human resources, knowledge, science and so on. Demand conditions mainly refer to the scale and demand of customers in the home market for products or services produced in the industry. Related and supporting industries are reflected in the production performance of upstream and downstream industries related to that industry. The strategy, structure and competition of enterprises are reflected in the organizational and management forms of enterprises. In terms of demand conditions, Porter emphasized the important role of the domestic demand market in driving economic growth, so it was measured by per capita consumption expenditure and per capita sales of social retail goods. Relevant supporting industries refer to the views of existing scholars, and use the proportion of the added value of the secondary and tertiary industries, which are closely linked to digital trade, to GDP respectively [16]. In terms of corporate strategy, corporate structure and peer competition, foreign investment and per capita e-commerce sales are selected to measure. Other indicators are selected as shown in Table 1. All relevant data are from the “2020 National Bureau of Statistics” and the statistical yearbooks of provinces and cities.

**Table 1:** Indicators for measuring the development level of digital trade based on diamond theory.

First order index	Secondary index	Three level index	ID
Factors of production	Human Resources	Information software and technology services as a percentage of total population employment	C1
		Registered urban unemployment rate	C2
		Illiteracy rate	C3
	Resource of knowledge	Number of newspapers and periodicals	C4
		Number of general institutions of higher learning	C5
		Number of legal entities in scientific research and technical services	C6
	Infrastructure	Internet broadband access port density	C7
		Internet penetration rate	C8
		Total per capita postal and telecommunications business	C9
		Road density	C10
		Long-distance cable line density	C11
		Number of legal entities in the cultural, sports and entertainment industry	C12
Corporate Strategy	Foundation of Enterprise	Total investment in foreign-invested enterprises	C13
		E-commerce sales per capita	C14
Conditions of demand	The domestic market	Per capita consumption expenditure of all residents	C15
		Total retail sales of consumer goods per capita	C16
Related supporting industries	Related supporting industries	Proportion of added value of secondary industry to GDP	C17
		Proportion of added value of tertiary industry to GDP	C18

### Qualitative comparative analysis

**Fig 2:**
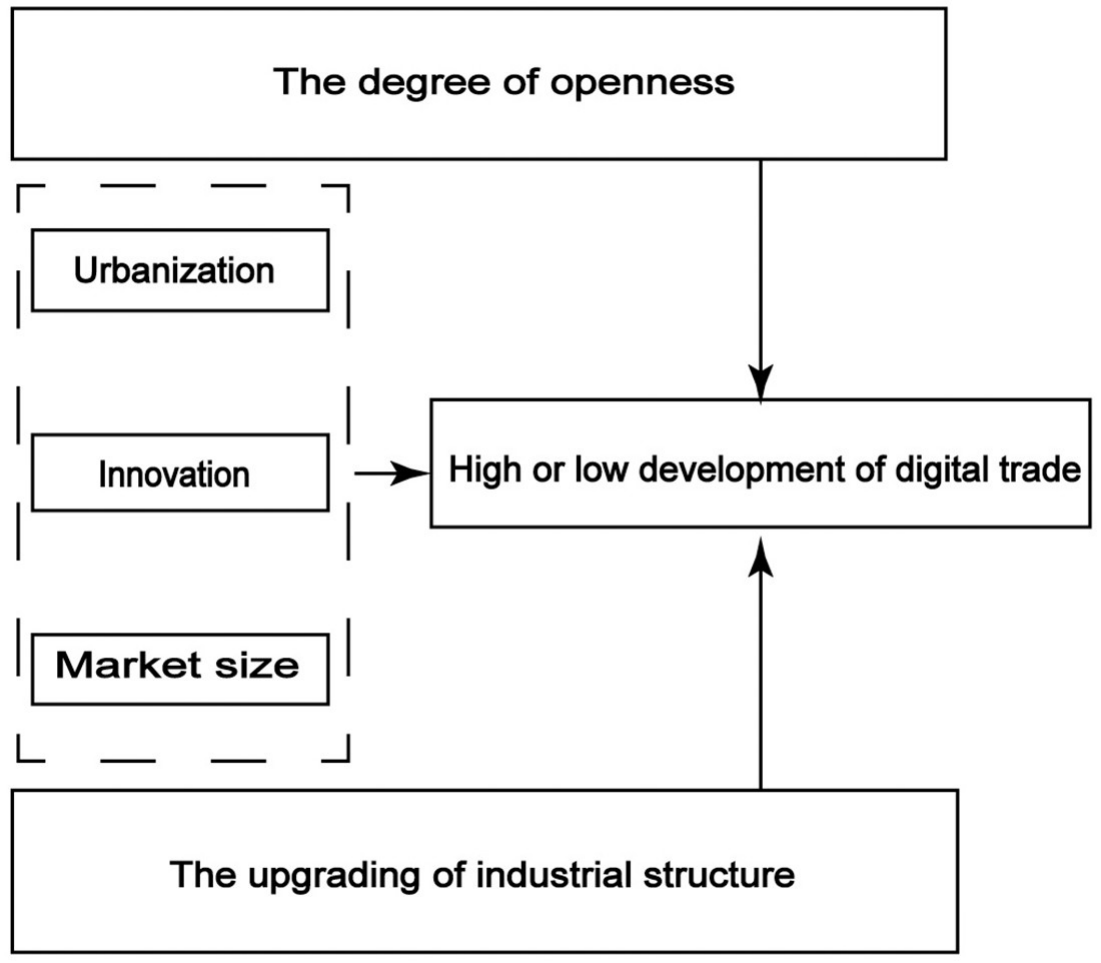
The logical framework for QCA.

The QCA method is based on sets, which is suitable for case studies of small and medium samples, as well as for the quantitative analysis of large samples, and the method breaks through some core assumptions of mainstream statistics, such as linear assumptions and causal symmetry, etc., in the analysis of medium samples (such as 10 to 40 cases), it is usually appropriate to choose 4 to 6 or 4 to 7 interpretation conditions, otherwise it is easy to lead to the emergence of limited diversity problems. The logical framework of QCA is shown in Fig 2. The development of digital trade is affected by many factors, and the most effective conditional variables to promote the development of digital trade are selected to the greatest extent. First, innovation. Innovation is the first driving force for vigorous economic development. Three indexes are selected: the number of legal entity in scientific research and technology service industry, the amount of technology market transaction, and the number of domestic patent applications [17]. The simple additive weighting is calculated to represent it by the entropy method mentioned above. Second, the size of the market. Market size can reflect the inherent potential of a region’s economic development. A higher level of market size can provide a broader space for the scale growth of digital trade. So we used regional per capita GDP to represent the regional market size [18]. Third, the level of urbanization. The increase in urbanization can attract more resources and talents, and has a significant promotion effect on economic growth [19, 20]. Therefore, the urbanization rate was calculated by calculating the proportion of the urban population to the permanent population at the end of the year [21]. Fourth, the upgrading of industrial structure. At present, China’s economic growth is mainly driven by the tertiary industry and the secondary industry [22]. The industrial structure is not only positively correlated with the growth of the regional economy [23], but also the optimization of industrial structure can also promote the high-quality development of the economy [24], so the ratio of added value of the tertiary industry and the secondary industry is selected to represent the upgrading of the regional industrial structure. Fifth, the degree of openness. The new development concept points out that opening up is crucial to the impact of national prosperity, so the degree of regional openness is measured by calculating the proportion of regional import and export trade to regional GDP. This paper focuses on the development of digital trade in 11 provinces and cities in the Yangtze River Economic Belt in 2020, which belongs to a medium sample size, so the selection of 5 conditional variables for analysis meets the premise of QCA. All relevant data are also derived from “the 2020 National Bureau of Statistics” and the statistical yearbooks of provinces and municipalities.

## Results

### Result of TOPSIS

Before starting the solution, the initial decision-making matrix must be constructed. The initial decision matrix of TOPSIS are shown in Table 2. The second column in Table 2 represents the results of Entropy method. All results are kept to three decimal places for subsequent analysis. In particular, it should be clarified that the initial decision matrix is transposed for page width.

**Table 2:** Weights and initial decision-making matrix.

ID	*W*_j_	Anhui	Zhejiang	Jiangsu	Shanghai	Hunan	Guizhou	Yunnan	Jiangxi	Hubei	Sichuan	Chongqing
C1	0.141	0.104	0.153	0.430	0.819	0.042	0.042	0.038	0.042	0.160	0.097	0.260
C2	0.004	0.385	0.279	0.340	0.294	0.198	0.287	0.279	0.340	0.325	0.303	0.242
C3	0.025	0.157	0.259	0.270	0.437	0.412	0.105	0.151	0.363	0.303	0.177	0.432
C4	0.024	0.303	0.385	0.501	0.268	0.325	0.121	0.147	0.200	0.232	0.416	0.170
C5	0.011	0.316	0.302	0.459	0.173	0.313	0.206	0.225	0.289	0.355	0.363	0.187
C6	0.068	0.232	0.417	0.733	0.151	0.227	0.055	0.132	0.109	0.287	0.193	0.097
C7	0.249	0.067	0.150	0.177	0.964	0.040	0.026	0.015	0.040	0.046	0.034	0.076
C8	0.003	0.258	0.343	0.313	0.426	0.274	0.294	0.263	0.251	0.263	0.283	0.306
C9	0.028	0.240	0.504	0.332	0.381	0.237	0.346	0.318	0.220	0.210	0.249	0.033
C10	0.014	0.350	0.242	0.306	0.422	0.236	0.243	0.154	0.262	0.324	0.168	0.455
C11	0.051	0.238	0.240	0.353	0.739	0.189	0.189	0.141	0.187	0.162	0.241	0.069
C12	0.034	0.246	0.559	0.504	0.145	0.294	0.107	0.192	0.153	0.281	0.285	0.183
C13	0.109	0.169	0.309	0.717	0.541	0.113	0.044	0.075	0.070	0.124	0.155	0.065
C14	0.179	0.101	0.185	0.153	0.936	0.062	0.004	0.049	0.072	0.087	0.070	0.179
C15	0.014	0.237	0.393	0.330	0.535	0.264	0.187	0.211	0.226	0.242	0.249	0.272
C16	0.042	0.264	0.362	0.385	0.564	0.215	0.179	0.183	0.202	0.276	0.010	0.323
C17	0.002	0.310	0.322	0.343	0.209	0.301	0.309	0.270	0.341	0.290	0.284	0.315
C18	0.002	0.282	0.311	0.290	0.410	0.284	0.282	0.284	0.269	0.293	0.291	0.295

The ranking results of each province are shown in Table 3. From the measurement results, Shanghai’s digital trade development is far ahead, far surpassing other provinces and cities, showing a trend of growth. Guizhou is at the bottom of the ranking. From the perspective of spatial distribution characteristics, the development of digital trade in the Yangtze River Economic Belt shows a trend of “high in the east and low in the west”, with the Yangtze River Delta region being the best. The measured results fully show that there is an imbalance and incoordination in the current development of digital trade in various provinces in the Yangtze River Economic Belt.

**Table 3:** The result of TOPSIS.

Area	S*	S^-^	C*	Rank
Anhui	0.297	0.034	0.103	5
Zhejiang	0.266	0.068	0.204	3
Jiangsu	0.248	0.117	0.322	2
Shanghai	0.047	0.317	0.871	1
Hunan	0.310	0.025	0.074	8
Guizhou	0.321	0.013	0.039	11
Yunnan	0.318	0.015	0.046	10
Jiangxi	0.311	0.020	0.059	9
Hubei	0.300	0.034	0.101	6
Sichuan	0.307	0.026	0.078	7
Chongqing	0.286	0.050	0.148	4

Regional economic differences will have a two-sided effect on economic development, which can not only enhance the enthusiasm and oppression of economic development in various regions, but also cause certain negative impacts. For example, the existence and change of regional economic differences will objectively aggravate the competitive behavior between regions, resulting in regional local protectionism and other phenomena, which is not conducive to the coordinated and sustainable development of the national economy to a certain extent. Moreover, widening disparities may lead to excessive flows of certain resources and factors of production from less developed regions, thereby limiting the development potential of the former. Underdeveloped regions, in turn, will restrict the latter’s economic development through market supply and demand due to the relative or absolute sluggish economic development. Therefore, in the future, the China should continue to promote the coordinated development of regional digital trade by improving relevant policies and solve the current problem of uneven development of digital trade in various regions of the Yangtze River Economic Belt.

### Result of QCA

Next is the QCA analysis. Considering that the study is for continuous variables, fsQCA is most appropriate. When analyzing with fsQCA, the variables must first be calibrated. Calibration must be done in conjunction with practical and theoretical calibration. On the basis of referring to the calibration method adopted by existing study [25], the upper quartile (fully affiliated), median (intersection), and lower quartile (completely non-affiliation) of each variable were selected for direct calibration of the variables, and the specific results are listed in Table 4. After variable calibration, in order to avoid being ignored because the membership of the case is at the intersection, the calibrated variable with a membership of 0.5 is adjusted by adding 0.001 to its membership degree below 1 [26]. After testing the necessity of the condition variables, it is found that the consistency level of all condition variables in a single condition variable does not constitute a necessary condition under the setting of a threshold of 0.9 [27].

**Table 4:** Results and condition variables calibrated.

Results and condition variables	Set of objects	Calibration		
		fully affiliated	intersection	completely non-affiliation
Level of development of digital trade	High level of development	0.204	0.101	0.059
The degree of openness	High level of openness	23.998	12.138	9.105
Market size	High market size	78173.000	62900.000	57065.000
The upgrading of industrial structure	Good industrial structure	1.456	1.338	1.293
Innovation	Driven by high-quality innovation	0.288	0.189	0.067
Urbanization rate	High level of urbanization	72.171	60.434	56.731

In the configuration analysis, referring to the existing research recommendations, the PRI consistency score is set to 0.75, and the consistency and case frequency thresholds are set to 0.8 and 1 respectively. The fsQCA software is used to generate three solutions, namely simple solutions, complex solutions and intermediate solutions. An intermediate solution is both a subset of a simple solution and a superset of a complex solution. Conditional variables that occur simultaneously in intermediate and simple solutions are called core conditions, and conditional variables that appear in intermediate solutions but do not appear in simple solutions are called supplementary conditions or edge conditions. Table 5 shows the results of the configuration analysis, which represents the high-level development configurations of digital trade in each province by performing Boolean simplification operations through intermediate solutions and simple solutions. According to the final configuration results, the consistency level of both the single solution and the overall solution is greater than the minimum requirement of 0.8. The overall coverage of the high development level configurations is 0.842, which is greater than the critical value of 0.5. From the perspective of all conditions for the development of digital trade in the Yangtze River Economic Belt, the market size and urbanization appear as core conditions in the two configurations, which shows that they are crucial to the development of digital trade in the provinces of the Yangtze River Economic Belt. However, as far as configuration C is concerned, only the degree of openness to the outside world as the core condition exists according to the high-level configuration results of digital trade. These three configurations can be summarized as “market size-urbanization” and “openness”. The paths fully demonstrate the importance of market scale and urbanization to the development of regional digital trade. Typical cases of these paths are Shanghai and Jiangsu. Yangtze River Delta is one of the strongest economic development regions in China. Strong information industry foundation, excellent communication infrastructure, sufficient market scale and other unique location conditions make it attract a lot of talents, material and financial resources, which provides superior conditions for the development of digital trade, so the current development level of digital trade in Jiangsu and Shanghai is more prominent.

**Table 5:** High-level configurations of digital trade in the Yangtze River Economic Belt.

Conditional variable	High development level configuration		
	Configuration A	Configuration B	Configuration C
The degree of openness	◇	○	◆
Market size	◆	◆	○
The upgrading of industrial structure	○	◇	●
Innovation		◇	◇
Urbanization rate	◆	◆	●
Consistency of solution	0.966	0.912	0.882
Original coverage	0.448	0.442	0.176
Unique coverage	0.298	0.321	0.072
Overall coverage	0.842		
Overall consistency	0.943		

Note:

^◆^ indicates the existence of core conditions,

^●^ indicates the lack of core conditions;

^◇^ indicates the existence of supplementary conditions,

^○^ indicates the lack of supplementary conditions.

Table 6 shows the results of the configuration analysis of the low-level development of digital trade. From the perspective of all conditions and configurations of digital trade development in the Yangtze River Economic Belt, the lack of market scale and urbanization level is the core condition for configuration E, configuration G and configuration H. Similarly, the configuration results are divided into “market size-urbanization-openness-innovation”, “market size-urbanization-innovation” and “market size-urbanization. These cases are represented by Guizhou Province and Yunnan Province. The reasons for the low level of digital trade development in these regions may be as follows: First, this paper uses per capita GDP to represent the size of the regional market [28], the survival of any economic activity must reach a certain demand scale to maintain its most basic operating costs, and the provinces with low level of digital trade development are also ranked at the bottom of their GDP, so the development space of digital trade in these provinces may be affected to a certain extent. This restricts the development of digital trade. Second, the improvement of urbanization can attract more excellent resources to the region, so the development of digital trade in areas with low urbanization level will lack effective power support.

**Table 6:** Low-level configurations of digital trade in the Yangtze River Economic Belt.

Conditional variable	Low development level configuration			
	Configuration E	Configuration F	Configuration G	Configuration H
The degree of openness	●	●	◇	◇
Market size	●		○	●
The upgrading of the industrial structure		◆	○	○
Innovation	●	●	◇	○
Urbanization	●	●	●	◇
Consistency of solution	1.000	1.000	0.999	1.000
Original coverage	0.603	0.313	0.286	0.172
Unique coverage	0.153	0.001	0.085	0.024
Overall coverage	0.712			
Overall consistency	0.999			

^◆^ indicates the existence of core conditions,

^●^ indicates the lack of core conditions;

^◇^ indicates the existence of supplementary conditions,

^○^ indicates the lack of supplementary conditions.

## Sensitivity analysis

**Fig 3:**
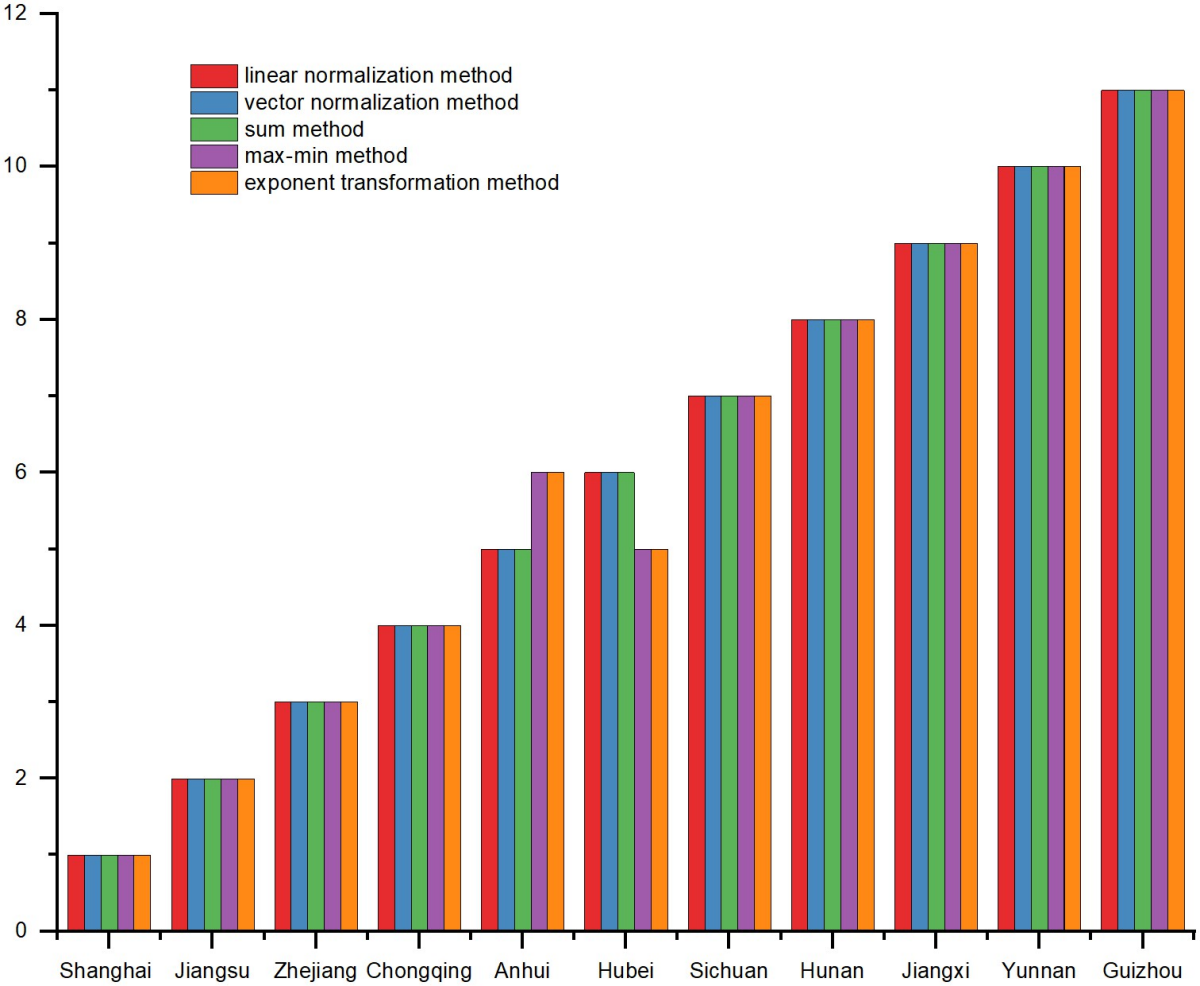
Ranking of areas using various normalization methods.

In order to ensure the reliability of the research conclusions, it is necessary to carry out sensitivity analysis. The most commonly used method is to change the weight coefficients [29]. We chose two ways to test whether the ranking results are robust when the weightings are changed. The first method was to follow the treatment of relevant scholars, changed one weight by 10%, while keeping the other weights unchanged [30]. In the end, no change was found in the regional rankings. The second method was to use vector normalization method (VNM), sum method (SM), max-min method (Max-min) and exponent transformation method (ETM) to standardize indicators, and determine the weights. Then, different weights and previous decision matrix were combined to calculate the ranking. The results were shown in Fig 3. It can be seen that when max-min method (Max-min) and exponent transformation method (ETM) are used, the rankings of Anhui and Hubei change. Shanghai’s ranking is firmly in first place, but Guizhou is still at the bottom of the ranking.

After obtaining the ranking results, we also calculated the Spearman rank correlations. Spearman rank correlations are used to measure the correlation between variables. It is calculated by the following formula:

$$\rho =1-\frac{6\sum_{i=1}^{m}D_{i}{}^{2}}{N(N^{2}-1)}$$ (13)

Where *ρ* stands for correlation coefficient, *D* for grade difference, *N* for sample size, and *i* for the number of evaluation objects. A correlation coefficient above 0.8 indicates a strong correlation between alternatives [31]. As shown in Table 7, at the significance level of 1%, the correlation values of each alternative scheme are between 0.991 and 1, which fully indicates that TOPSIS has been well implemented in solving the problem.

**Table 7:** Spearman rank correlations and Wilcoxon rank tests.

Coefficient	LNM	VNM	SM	Max-min	ETM
LNM	1	1	1	0.991**	0.991**
VNM		1	1	0.991**	0.991**
SM			1	0.991**	0.991**
Max-min				1	1
ETM					1

Note:

** represents the significance level of 1%.

**Fig 4:**
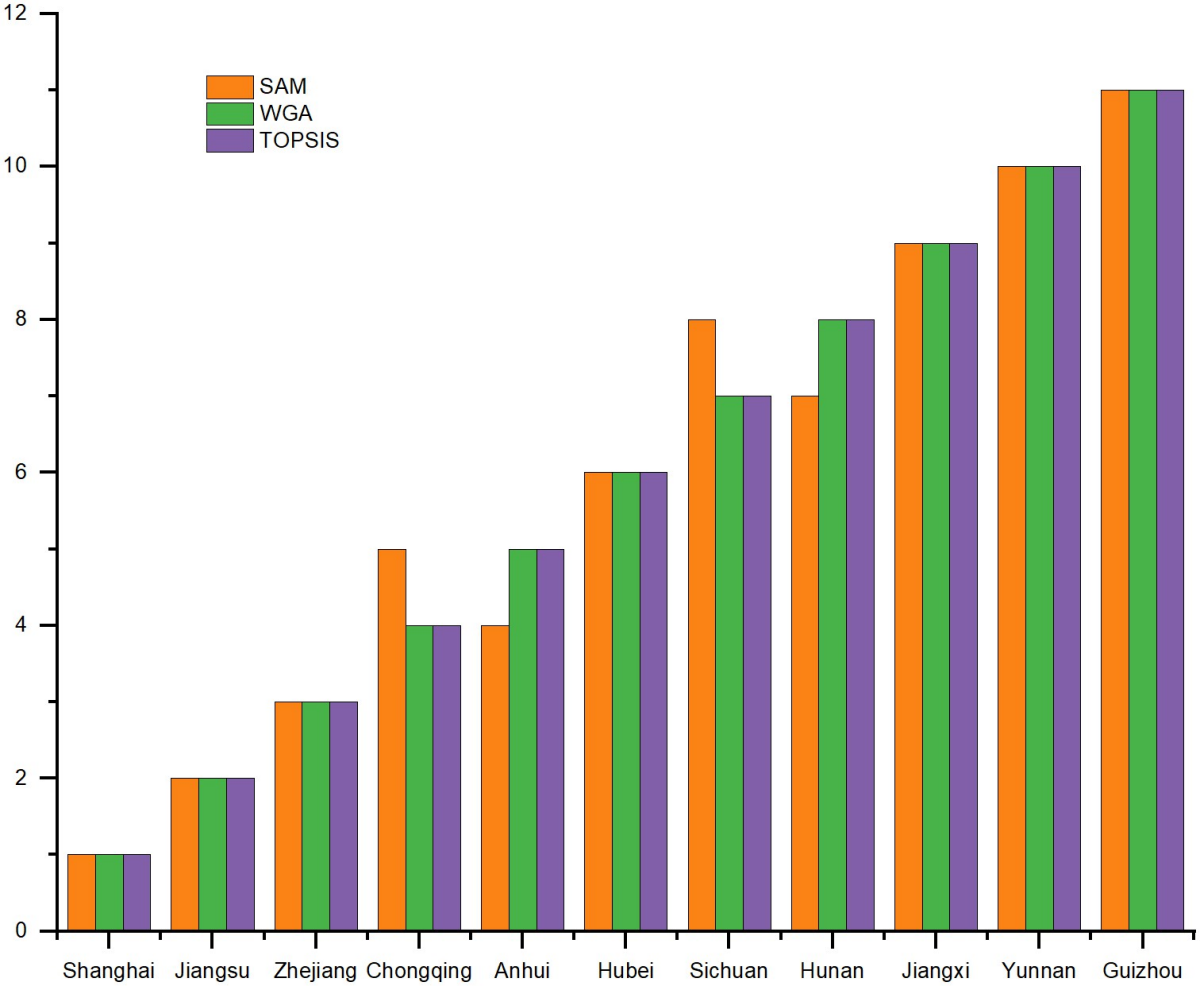
Ranking of areas using various MCDM methods.

Finally, the reliability of the results was also verified by changing the comprehensive evaluation method with reference to the relevant literature [32]. Specifically, simple additive weighting (SAW) and weighted geometric average (WGA) were selected for calculation. The final ranking was shown in Fig 4. As can be seen from the figure, the rankings of Shanghai, Jiangsu and Zhejiang have not changed, which is consistent with the above analysis results. However, when SAW method is used, the ranking of Hunan, Anhui, Chongqing and Sichuan have changed.

Similarly, Spearman rank correlations are shown in Table 8. At the significance level of 1%, the correlation values of each alternative scheme are between 0.982 and 1, which also indicates that TOPSIS has been well implemented. To sum up, the entropy method and TOPSIS are used in this study to solve the region ranking problem well, and the ranking results are robust and reliable. In all scenarios, the ranking of Shanghai, Jiangsu and Zhejiang remained unchanged, with Shanghai first, Jiangsu second and Zhejiang third. The analysis also showed that Guizhou ranked the lowest, followed by Yunnan and Jiangxi, which did not change their rankings. Anhui Province had the most frequent changes in ranking, followed by Hubei Province.

**Table 8:** Spearman rank correlations and Wilcoxon rank tests.

	SAM	WGA	TOPSIS
SAM	1	0.982**	0.982**
WGA		1	1
TOPSIS			1

Note:

** represents the significance level of 1%.

## Conclusions and discussion

China’s digital trade is growing rapidly, but uneven development between regions still exists. Taking the Yangtze River Economic Belt as an example, this paper analyzes the development level of digital trade in each region by means of entropy method and TOPSIS. Besides, this paper uses QCA to discuss the core factors affecting the development of digital trade. Finally, the robustness of the research conclusion is tested. According to the results of entropy weight TOPSIS, the development of digital trade shows a large regional imbalance and uncoordinated development. The Yangtze River Delta region has the highest level of digital trade development, especially Shanghai, showing a trend of growth. The digital trade in Shanghai, Jiangsu and Zhejiang is the most developed, ranking the top three all the time. We mainly changed the weightings and evaluation method to investigate the robustness of the conclusion. And no matter how we change the method of sensitivity analysis legitimately, Guizhou and Yunnan always rank in the bottom two (S1 File). Spearman rank correlations also show that our study is reliable.

In addition, the level of urbanization, degree of openness, market size, upgrading of industrial structure and innovation cannot alone constitute the necessary conditions for the development of digital trade. There are two configuration paths for the high development level of digital trade and there are three main paths that constitutes the low development level of digital trade. What’ more, Market scale and urbanization appear most frequently in the configurations, so in order to promote the coordinated development of regional digital trade, it is not possible to ignore the promotion of urbanization level and market scale in underdeveloped areas. In order to effectively narrow the differences in digital trade between provinces in the Yangtze River Economic Belt in the future, the government should continue to formulate measures to narrow regional disparities. For underdeveloped areas, the government can increase investment in infrastructure, and give priority to the layout of key projects in underdeveloped areas. To a certain extent, the differences in the promotion of China’s economic system reform have led to the uneven degree of economic marketization between regions, so the coordinated management system and operation mode between regions are the basic requirements for achieving coordinated regional economic development. All relevant departments should also attach importance to the cultivation of digital technology talents, establish the goal of training digital trade talents, improve the construction of digital trade talent training system, and provide guarantee for the development of digital trade in the Yangtze River Economic Belt and even the whole country.

Although the development level of digital trade in the Yangtze River Economic Belt is measured on the one hand, the influencing factors and improvement paths of digital trade on the other hand, there are still some shortcomings. Firstly, due to the difficult availability of data, this paper takes the provinces of the Yangtze River Economic Belt as the research perspective, and the conclusions drawn may not be applicable to more micro level, such as cities or enterprises. Secondly, this paper takes TOPSIS and QCA as research methods. Although the key factors affecting the development of digital trade are clarified, it does not clarify to what extent these factors can affect it. In the future, we can try to establish a model combining NCA (Necessary Condition Analysis) and QCA to further explore the specific influence of these factors. The logic of QCA is mainly adequacy analysis, while the logic of NCA is to explore the necessary inadequacy of a single variable. The NCA has been put forward for a short time, and the literature on the combination of these two methods is still relatively few.

## Supporting information

S1 File (PDF)
