# Supplementary material for: Research on the influencing factors and improvement paths of digital trade development in the Yangtze River Economic Belt——Based on entropy-weighting TOPSIS and fsQCA method
Source: PLoS One. 2023 Jul 17;18(7):e0284519. doi: 10.1371/journal.pone.0284519 (PMC10351719; doi:10.1371/journal.pone.0284519)
Supplement: S1 File — (PDF) [file pone.0284519.s001.pdf]

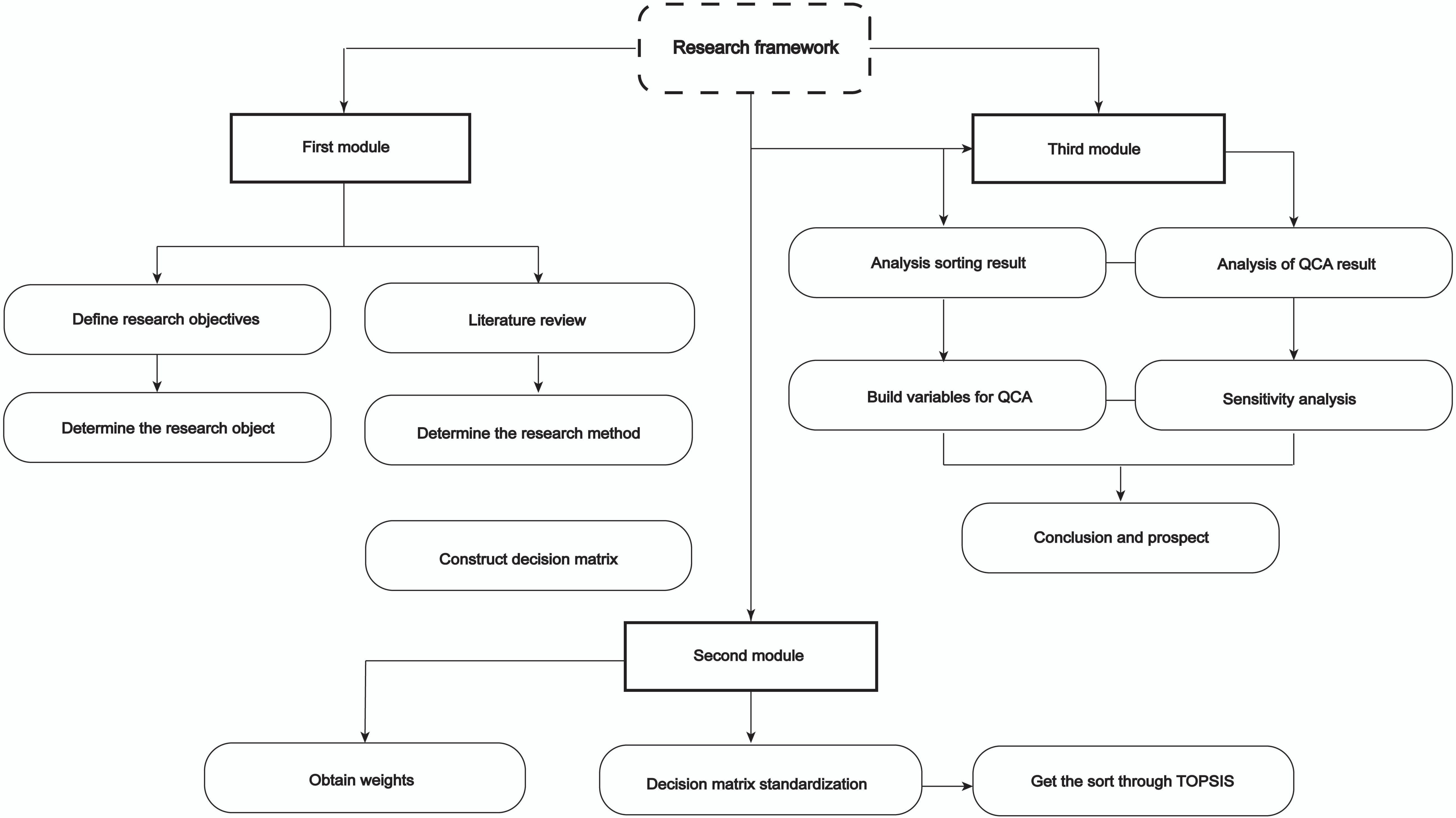
Research framework
First module
Third module
Analysis sorting result
Analysis of QCA result
Define research objectives
Literature review
Build variables for QCA
Sensitivity analysis
Determine the research object
Determine the research method
Conclusion and prospect
Construct decision matrix
Second module
Obtain weights
Decision matrix standardization
Get the sort through TOPSIS

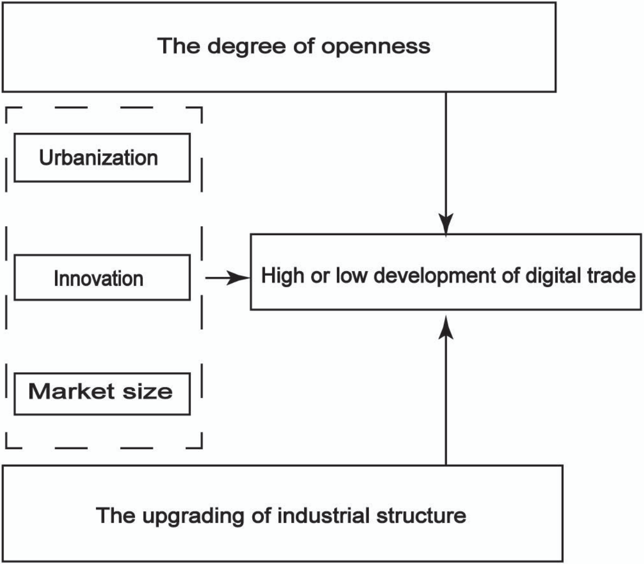
The degree of openness
Urbanization
Innovation
Market size
High or low development of digital trade
The upgrading of industrial structure

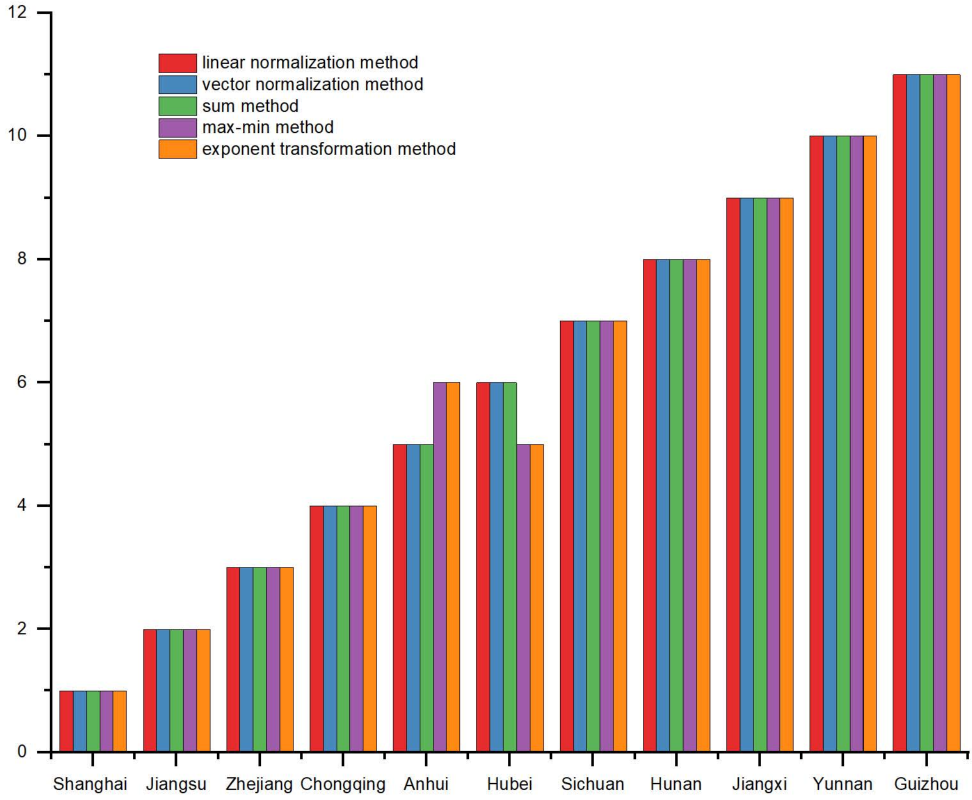

linear normalization method
vector normalization method
sum method
max-min method
exponent transformation method
0
2
4
6
8
10
12
Shanghai
Jiangsu
Zhejiang
Chongqing
Anhui
Hubei
Sichuan
Hunan
Jiangxi
Yunnan
Guizhou

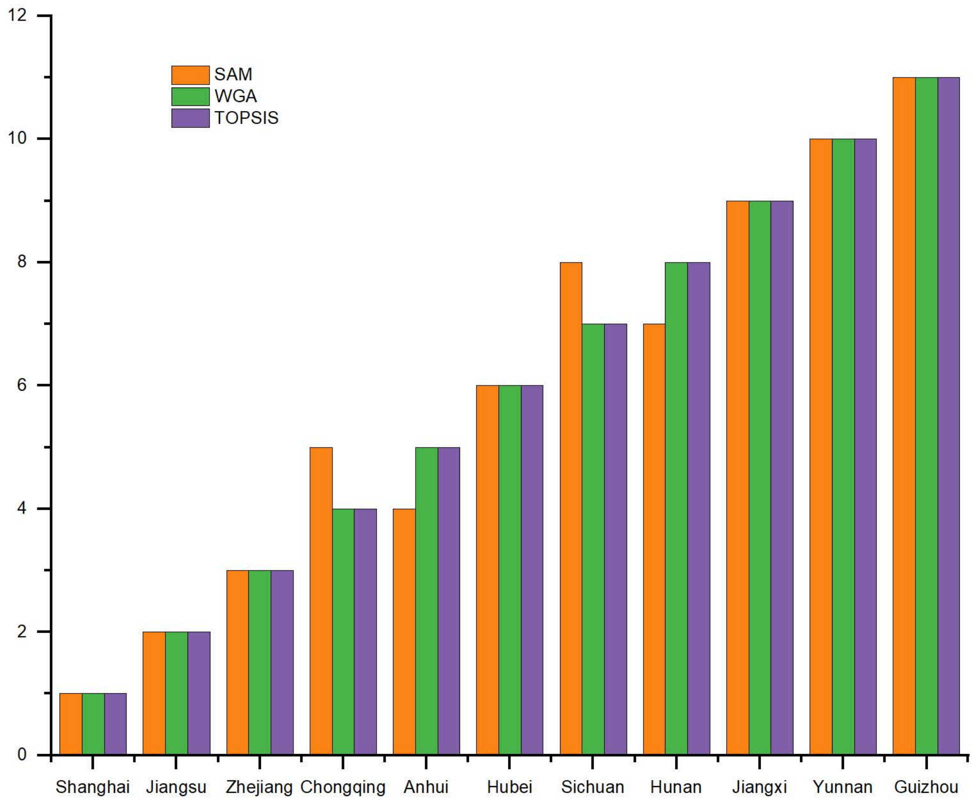

SAM
WGA
TOPSIS
0
2
4
6
8
10
12
Shanghai
Jiangsu
Zhejiang
Chongqing
Anhui
Hubei
Sichuan
Hunan
Jiangxi
Yunnan
Guizhou
